# Cognitive reserve and dementia A scientometric review

**DOI:** 10.1590/1980-57642018dn13-010001

**Published:** 2019

**Authors:** Maria Helena Pestana, Margarida Sobral

**Affiliations:** 1 PhD, University Institute of Lisbon (ISCTE-IUL), Lisbon, Portugal. Research and Education Unit on Ageing (UNIFAI, ICBAS, UP).; Universidade do Porto, Instituto de Ciências Biomédicas Abel Salazar, Unidade de Investigação e Formação sobre Adultos e Idosos, Portugal; 2 PhD, Psychogeriatrics Service, Hospital Magalhães Lemos, Porto, Portugal. Research and Education Unit on Ageing (UNIFAI, ICBAS, UP). CINTESIS - Center for Health Technology and Services Research (FM, UP).; Universidade do Porto, Instituto de Ciências Biomédicas Abel Salazar, Unidade de Investigação e Formação sobre Adultos e Idosos, Portugal; Universidade do Porto, Faculdade de Medicina, Center for Health Technology and Services Research, Portugal

**Keywords:** cognitive reserve, dementia, intellectual structure, patterns, emerging trends, CiteSpace, reserva cognitiva, demência, estrutura intelectual, padrões, tendências emergentes, CiteSpace

## Abstract

Research into cognitive reserve (CR) and dementia is advancing rapidly. This paper analyses the intellectual structure, emerging trends and relevant shifts in the development of available knowledge. Data collected from the Web-of-Science produced an expanded network of 564 articles and 12,504 citations in the 1998-2017 period. The co-citation network visualized was characterized by a scientometric review using CiteSpace. The results revealed that author Stern Y had the highest number of publications and citations. The network of journals, institutions and countries showed a central-peripheral structure with Neurology, Harvard University and the USA ranked first, respectively. While cognitive reserve remains the most prominent area of research in this field, studies related to functional ability, executive control, mortality data and reserve mechanisms have grown considerably. The identification of critical articles and the development of emerging trends highlights new insights in the area of research, better communicating key findings and facilitating the exploration of data.

## RESUMO.

A pesquisa em reserva cognitiva e demência avança rapidamente. Este artigo analisa a estrutura intelectual, tendências emergentes e mudanças relevantes no desenvolvimento do conhecimento coletivo. Dados coletados do Web-of-Science, construíram uma rede expandida de 564 artigos e 12.504 citações, entre 1998-2017. A rede de cocitação visualizada foi caracterizada através de uma revisão cientométrica utilizando o CiteSpace. Os resultados revelam Stern Y com o maior número de publicações e citações. A rede de revistas científicas, instituições e países apresenta uma estrutura central-periférica, respetivamente com Neurologia, Universidade de Harvard e EUA em primeiro lugar. Enquanto a reserva cognitiva continua a ser a área de pesquisa mais proeminente neste campo, estudos relacionados à capacidade funcional, controle executivo, dados de mortalidade e mecanismos de reserva têm experimentado um crescimento considerável. A identificação de artigos críticos e o desenvolvimento de novas tendências emergentes destacam novos insights sobre a área de pesquisa, comunicando melhor as principais descobertas e facilitando a exploração de dados.

Due to the progressive ageing of the population and life expectancy, increasing attention has been dedicated to the study of cognitive reserve (CR) and dementia. Dementia predominantly affects older people, and the risk of dementia rises with increasing age. Alzheimer’s disease (AD) is the most common form of dementia in the elderly.^1^ CR is a hypothetical model which reflects cognitive aging and describes the capacity of the adult brain to tolerate the effects of this neurodegenerative process,^2^ while dementia is a syndrome in which there is deterioration in memory, thinking, orientation, comprehension, calculus, learning capacity, language, and judgement, thinking, behavior and the ability to perform everyday activities. The CR hypothesis suggests that individual differences in the ability to cope with AD pathology^2^^-^ 5 are consistent with the prediction that people with greater reserve can cope with advancing AD pathology longer before it is expressed clinically.^2^^,^ 5 ^-^ 7 CR is not fixed and continues to evolve throughout the lifespan.^6^^,^ 7 CR is not measured directly^8^ and the variables that pertain to lifetime experience, such as education, occupation, and leisure activities, are the most commonly used proxies for CR.^2^^,^ 6 ^,^ 8 ^-^ 10 These variables help the individual retain cognitive function in old age.^11^ Some studies have shown that the risk of developing AD is reduced in individuals with higher levels of education,^5^^,^ 12 ^-^ 16 occupational attainment^5^^,^ 17 ^-^ 21 and participation in leisure activities.^5^^,^ 22 ^-^ 26 Other studies found no association between education and incident dementia.^15^^,^ 27 ^,^ 28 Furthermore, no association was found between occupational attainment and incident AD in several population-based longitudinal studies.^29^ CR interventions might be a key nonpharmacological approach to preventing this disease.^6^ Several studies on CR and dementia have used different methodologies, leading to disparate results.

CR and dementia involve research in several fields and disciplines such as clinical neurology, neurosciences, geriatrics and gerontology. As research in these areas advances rapidly, it is critical to keep abreast of the respective intellectual structure, emerging trends and relevant shifts in the development of available knowledge. Studies on CR and dementia have been published in a large number of journals by authors from all over the world. However, there is a need to gather systematic data on this global scientific output. Therefore, innovative studies are required to make sense of the subsequent ballooning number of publications in the area. In recent years, bibliometrics has been widely applied in various fields to identify the output of authors, institutions and countries, as well as evaluate geographic distributions and international collaborations.^30^ In this study, bibliometric analysis was used to evaluate the global scientific output on CR and dementia, and find an approach to quantitatively and qualitatively assess the current global research. Data were based on the science citation index expanded database from the Institute of Scientific Information Web of Science database, corresponding to 564 publications and 12,504 citations referring to cognitive reserve and dementia in the 1998-2017 period. More specifically, this study aimed to analyze publications, journals, areas of research, the intellectual structure, keywords, research hotspots and emerging trends in CR and dementia.

## METHODS

### Network analysis and visualization

Bibliometric analysis is critical for conducting periodic reviews of existing research fields, identifying contributions to knowledge and for constructing substantiated arguments about the development of a field.^31^ The present study focused on co-citation analyses to create network visualizations of the relationships between influential publications, highlighting disciplinary contributions in an interdisciplinary field.^32^ CiteSpace was used to support network visualization from bibliographical sources and to quantify the science research using three types of metrics: (1) structural (betweenness centrality, modularity and silhouette), temporal (citation burst) and semantic (define cluster labels from phrases extracted from titles, abstracts and keywords or from index terms of citing articles). These metrics are useful for describing the intellectual structure of disciplines and fields, detecting existing scientific schools and academic networks, and for identifying potential research fronts, as will be seen later in the paper.

### Bibliographical records

The global scientific outputs were generated from the Web-of-Science (WoS) database and analysed using CiteSpace (http://cluster.cis.drexel.edu/~cchen/citespace/) and VosViewer (http://www.vosviewer.com/). The dimensions used as a basis for selecting the articles on cognitive reserve and dementia were: keywords, journals and years of publication. Concerning keywords, given the focus on cognitive reserve and dementia, these two words were used. To provide more scientific and accurate information about our research, only those articles with the search terms in the title, abstract or keywords of the documents were extracted for further analysis^33^ from the WoS database. Regarding time horizon, the analysis spans twenty years, from 1998 to 2017. Many previous articles have adopted a similar time horizon, e.g. Ye et al.^34^ Finally, concerning the journals, the number of selected papers explicitly focused on cognitive reserve and dementia was taken into account. Only journals published in English were included in the sample. Using these three criteria (keywords, time horizon and journals), the total sample included 564 articles, representing 82.10% of all the documents, and 12,504 citations. Based on the assumption that the citing of an article makes it relevant to the topic,^30^ articles not cited by other studies and therefore disconnected, were eliminated. According to Ohba et al.,^35^ findings on highly cited articles are useful to reveal the recognition of scientific advancement and give a historic perspective on scientific progress. Citation, as an association-of-ideas index, offers an approach to subject control of the literature of science.^36^ Some papers are not relevant for a specific research stream, they are not cited by other studies and therefore remain “disconnected”. Exclusion of disconnected articles from the overall sample, gave a final sample of 528 connected articles. These articles were distributed across 256 journals on the field of research, and 27 had no citations. The other 89.45% with at least one citation are distributed into a network with reasonable quality (modularity 0.4295) and low density (0.0258), suggesting they are dominated by a small number of relevant journals. The top 10 journals in the WoS represent 37.25% of all CR and dementia journals and accounted for 23.94% of total publications. *Neurology* and *Journal of Alzheimer Disease* accounted for most of research, totaling 51 documents (7.98%), followed by *Journal of the International Neuropsychological Society, Dementia and Geriatric Cognitive Disorders, International Journal of Geriatric Psychiatry, International Psychogeriatrics, American Journal of Geriatric Psychiatry, Journal of the American Geriatric Society*, and *Neurobiology of Aging*.

**Figure 1:**
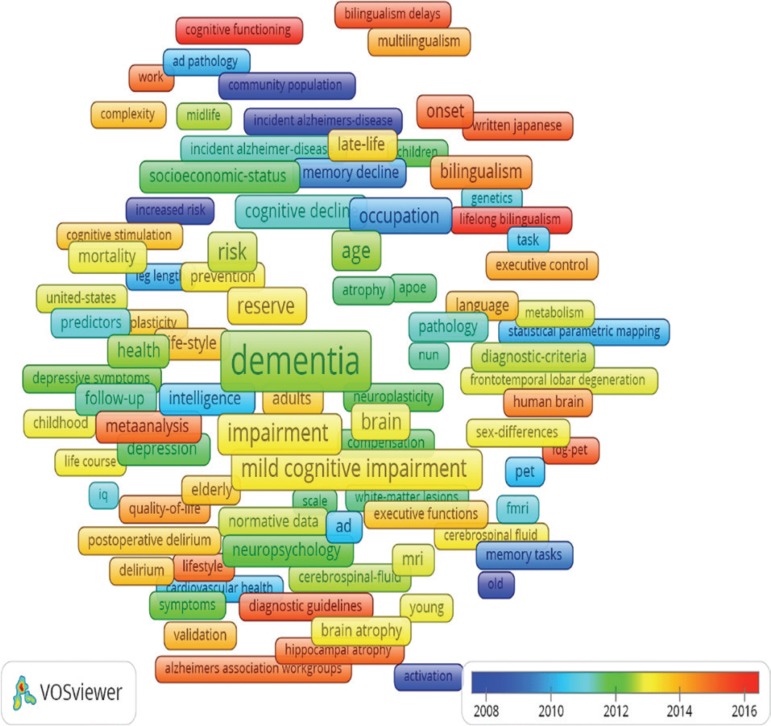
Network of keywords (1997-2018).

Source: authors from VosViewer.

The network has 2365 keywords, where 285 had the minimum number of five occurrences, and the top ones are shown by time in Figure 1. The more recent key words are marked in red, such as diagnostic guidelines, human brain, lifelong bilingualism, life style, and prevention.

The structure of the network contains 1043 institutions and is dominated by a small number of them (very low density 0.00142). At the top, institutions with more than 20 documents include: Harvard University, with 23 documents and 1504 citations, followed by Colombia University, with 26 documents and 4003 citations, and finally UCLA, with 23 documents and 653 citations. All these Institutions are in the USA.

The sample of connected articles shows a pattern of increasing publications (Figure 3), with a relative growth rate of 0.28 per year. The number of research publications is doubling every 3.28 years, which is an indicator of relatively rapid growth in the amount of research work being done on CR and dementia over the last twenty years.

**Figure 2:**
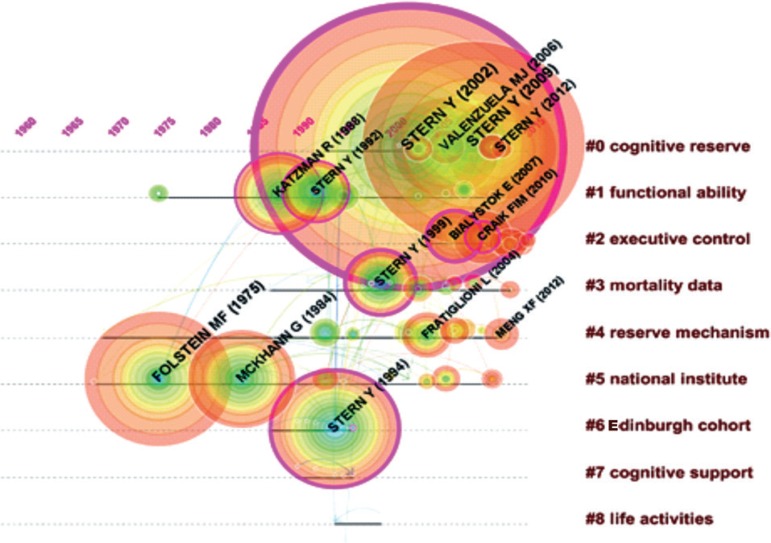
Timeline of co-citation clusters. Landmark articles are labeled.

**Figure 3:**
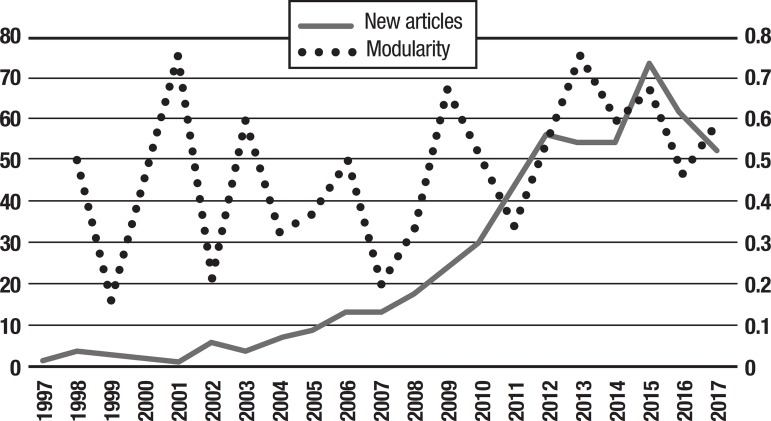
Structural changes in the network measured (1998-2017)

## BIBLIOGRAPHICAL LANDSCAPE

### Thematic clusters

Co-citation analysis is a useful empirical technique for describing the intellectual structure of disciplines.^37^ This technique uses pairs of documents which often appear together in reference lists and have something in common.^38^ CiteSpace divides the co-citation network into many clusters of co-cited references such that references are tightly connected within the same clusters, but loosely connected among different ones. This methodology focuses on references and, in this sense, explores the pillars of a specific research stream. Chen et al.^39^ recommended that the number of relevant clusters in the network should have more than 10 members and a silhouette value >0.70, which is an indicator of its homogeneity or consistency, indicating good quality of the clustering configuration.

The network generated 60 clusters (#). The pattern of our network is dominated by small clusters (low density 0.0098), with clear boundaries (silhouette >0.70), such that nodes within the same group are connected tighter than nodes between different groups (high modularity 0.9549). Only seven clusters have at least 10 members and are highly homogeneous (Table 1). Each cluster is labelled by noun phrases from titles of citing articles of the cluster,^40^ through three different algorithms: term frequency*inverse document frequency (Tf*idf), mutual information tests (MI), and log-likelihood ratio (LLR), where this last algorithm usually yields the best result in terms of uniqueness and coverage.^41^

**Table 1:** Major clusters of co-citation references.

Cluster	Size	Silhouette	Label (TF*IDF)	Label (LLR)	Label (MI)	Avg. Year
0	26	0.642	Underlying cognitive reserve	Cognitive reserve	Elderly adult	2004
1	19	0.739	Mental status | preclinical Alzheimer's disease	Functional ability	MRC CFA	1999
2	18	0.988	Information | history	Executive control	Poststroke aphasia	2011
3	17	0.777	Sex difference | clinical severity	Mortality data	MRC CFA	2000
4	16	0.623	Japanese population | identification	Reserve mechanism	Ethnic variation	2002
5	15	0.602	Reversion	National institute	Metabolic syndrome	1998
6	12	0.949	Neuropsychological assessment	Edinburgh cohort	Cognitive reserve	1993

Source: authors (2018) from CiteSpace.

The recentness of a cluster is indicated by the average year of publication. For example, cluster #2 has an average year of 2011 and is the most recently formed cluster, while clusters #6 have an average year of 1993, representing the oldest clusters, as well as the least representative cluster with only twelve references.

The title of the top references that most cite the articles included in a cluster are marked in italics and can shed some light on the research done in that cluster. The largest cluster (#0) has 26 members, and is labelled *cognitive reserve* by LLR. The most active citer of #0 covering 19% of their references, is Perneczky et al.,^42^ on “*Cognitive reserve* and its relevance for the prevention and diagnosis of dementia”. According to these authors, progressive brain damage is undoubtedly the main cause of clinical symptoms of dementia in neurodegenerative disorders such as AD. However, the association between brain damage and cognitive symptoms is not linear. Certain interindividual differences such as a good school education or a greater brain volume are associated with a higher resilience against brain damage, usually referred to as CR. In subjects suffering from progressive neurodegeneration, active mechanisms, mechanisms that are associated with the ability to maintain a certain level of cognitive performance in the face of progressive neurodegeneration for a longer period, help to compensate for brain damage. The article focusses on the positive association between CR and the active mechanisms that contribute to the adaptation of brain activity when task difficulty level is increased. Two articles account for 15% of cluster #0 references: Bartres-Faz and Arenaza-Urquijo,^43^ on “Structural and functional imaging correlates of *cognitive* and brain *reserve* hypotheses in healthy and pathological aging”, focusses on brain plasticity and on a complex correspondence between active and passive components of reserve; and Whalley et al.,^44^ on “*Cognitive reserve* and the neurobiology of *cognitive* aging”, focuses on the association between CR and lifestyle choices (early and mild), early education, lifelong dietary habit, leisure pursuits and the retention of late life mental ability.

The second largest cluster (#1) has 19 members and is labelled *functional ability* by LLR. The most active citers for this cluster are Roe et al.,^45^ making up 26% of #1 references in “Alzheimer disease identification using amyloid imaging and reserve variables proof of concept”, focusing on the importance of the factors that influence AD pathology and dementia, to improve the predictive accuracy of amyloid imaging; and Geerlings et al.^46^ covering 21% of #1 references in “Cognitive reserve and mortality in dementia: the role of cognition, *functional ability* and depression”, focusing on the positive association between higher CR and mortality rates when clinical symptoms are more severe.

The third largest cluster (#2) has 18 members, is the newest cluster, with an average publication year of 2011, and is labeled *information* by TF*IDf and *executive control* by LLR. The most active citers are Guzman-Velez and Tranel,^47^ in “Does bilingualism contribute to cognitive reserve? Cognitive and neural perspectives” and Perani and Abutalebi,^48^ in “Bilingualism, dementia, cognitive and neural reserve”, both accounting for 33% of #2 references. These articles focus on the relationship between CR and bilingualism *information*.

The 4th largest cluster (#3) has 17 members and is labeled *mortality* data by LLR. The most active citers are Katzman,^49^ in “Epidemiology of Alzheimer’s disease and dementia: advances and challenges”, focusing on the age dependency of dementing disorders; and Qiu et al.,^50^ in “The influence of education on clinically diagnosed dementia incidence and *mortality* data from the kungsholmen project”, focusing on aging of the population and on the positive association between level of education and AD or dementia. No effect was found between education and mortality. Both citers made up 35% of the references included in #3.

The 5th largest cluster (#4) has 16 members and is labelled *reserve mechanism* by LLR. The most active citers for this cluster are Borroni et al.^51^ in “*Reserve mechanisms* in neurodegenerative diseases: from bench to bedside and back again”, focusing on novel therapeutic targets in neurodegenerative diseases; Daffner^52^ in “Promoting successful cognitive aging: a comprehensive review”, exploring the positive association between CR and enhancing brain capacity; and Jones et al.,^53^ in “Aging, brain disease, and *reserve*: implications for delirium”, centered on the prevention strategies for delirium and its role in aging and neuropsychiatric disease. All these citers together comprise 25% of #4 references.

Another major cluster corresponds to *reversion, or metabolism syndrome* (cluster #5). The most active citers are Kawano et al.,^54^ covering 20% of # 5 references in “Effects of educational background on verbal fluency task performance in older adults with Alzheimer’s disease and mild cognitive impairment”, investigating the importance of subjects’ educational background to analyse the effect of fluency task on the risk for developing AD; and Bruandet et al.^55^ accounting for 13% of #5 references in “Cognitive decline and survival in Alzheimer’s disease according to education level”, addressing the rate of cognition declines and survival in AD.

Finally, cluster #6 labelled *neuropsychological assessment*, or *Edinburgh cohort,* has an average publication year of 1993. The most active citers are Basso and Bornnstein^56^ in “Estimated premorbid intelligence mediates neurobehavioral change in individuals infected with HIV across 12 months”, focusing on the hypothesis that estimated premorbid intelligence mediates decline in neuropsychological function in patients with stable HIV status, and Pereda et al.^57^ in “Factors associated with *neuropsychological* performance in HIV-seropositive subjects without aids”, on the effect of not being on zidovudine treatment, having lower reserve capacity and being of older age, to lower the threshold for neuropsychological abnormalities in cases of early HIV infection. All these citers cover 50% of #6 references.

### The intellectual structure of CR and dementia

CiteSpace characterizes emerging trends and patterns of change in terms of visual attributes. Each node is depicted with a series of tree-rings across the time slices. The purple ring displays the structural properties of a node. The thickness of the purple ring displays the degree of betweenness centrality, which measures the transformative potential of a scientific study, essential for the development of a knowledge domain. The size of the node indicates the strength of citations the reference received. The color of the citations rings indicates the time slices in which the citation burst occurs. Red colours indicate more recent slices while blue colours indicate the opposite.

Figure 2 shows a timeline visualization of the network with distinct clusters, where some landmark articles are identified by first author. Cluster #2 has the higher number of references with a burst of citations in 2017, showing the relevance of *executive control* research to the current study of CR and dementia. Nevertheless, Stern^5^ from cluster #0 has the highest burst of citations in 2017 of all networks, showing its dominance for the current study of *cognitive research*.

The most cited papers have value in their revelation of advances in theory-building, and in providing a historical perspective on the progress made within a discipline.^4^ They are usually regarded as landmarks because of their ground-breaking contributions. The five top-cited articles are distributed between two areas of research (#0 and #4) between 1998-2017 (Table 2). At the top, are Stern^5^^,^ 58 ^,^ 59 and Katzman^60^ from cluster #0, respectively with 2570, 1776, 1242, and 1036 citations; and Fratiglioni et al.,^23^ from cluster #4 with 1636 citations. Stern^5^^,^ 58 ^,^ 59 develops a coherent theoretical background of reserve and CR, and focuses on the relevance of lifelong experiences to increasing CR, including educational and occupational attainment, and leisure activities in later life. Katzman^60^ focused on the effect of education on being diagnosed with dementia at an earlier point in time. Fratiglioni et al.^23^ focus on the importance of an active and socially integrated lifestyle in late life to protect against dementia and AD.

**Table 2:** Top-cited articles on Cognitive Reserve and Dementia.

Citations	Title	Author	Year	Centrality	Source	#
2570	What is cognitive reserve? Theory and research application of the reserve concept	Stern	2002	0.63	Journal of the International Neuropsychological Society	0
1776	Cognitive Reserve	Stern	2009	0.05	Neuropsychologia	0
1636	An active and socially integrated lifestyle in late life might protect against dementia	Fratiglioni et al.	2004	0.03	Lancet Neurol	4
1242	Education and the prevalence of dementia and Alzheimer's disease	Katzman	1993	0.18	Neurology	0
1036	Cognitive reserve in ageing and Alzheimer's disease	Stern	2012	0.03	Lancet Neurol	0

Source: authors (2018) from CiteSpace.

The most cited and betweenness centrality papers play an important role in different fields of CR and dementia research. Betweenness centrality articles indicate their importance in bridging different stages of field development.^61^ Stern Y is an author highly cited and belongs to three different clusters (#0, #1, #3), suggesting centrality in connecting these areas of research, contributing to the transformative improvement in CR and dementia research. Stern^58^ has the highest level of betweenness centrality (0.63). Another central article is Bialystok et al.,^62^ from cluster #2, connecting #2 (executive control) with #0 (cognitive reserve), currently highly cited, with a citation burst from 2013 to 2017 (Table 3). This article addressed the effect of lifelong bilingualism on dementia, which maintains cognitive functioning and delays the onset of symptoms of dementia in old age by about 4 years. Stern^58^ and Bialystok et al.,^62^ are also articles that have contributed to a structural change in the network, in 2002 and 2007, respectively (Figure 3).

**Table 3:** Structurally and temporally significant references.

Author references	#	Source	Year	Centrality	Strength	Begin	End	1998 - 2017
Stern	0	Lancet Neurol	2012	0.03	242.959	**2014**	2017	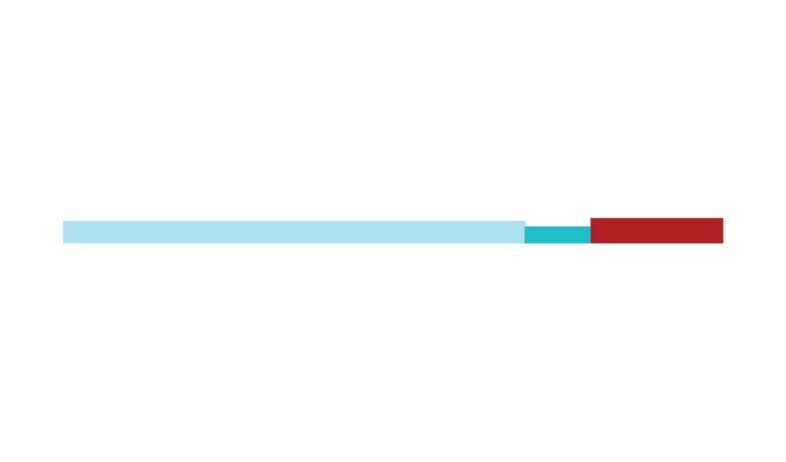
Katzman	0	Neurology	1993	0.18	138.902	**2002**	2010	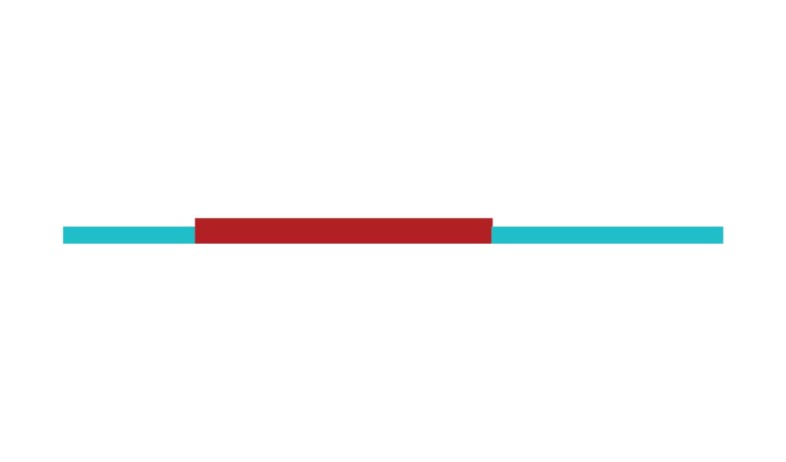
Tucker and Stern	0	Curr Alzheimer Res	**2011**	0.01	99.507	**2014**	2017	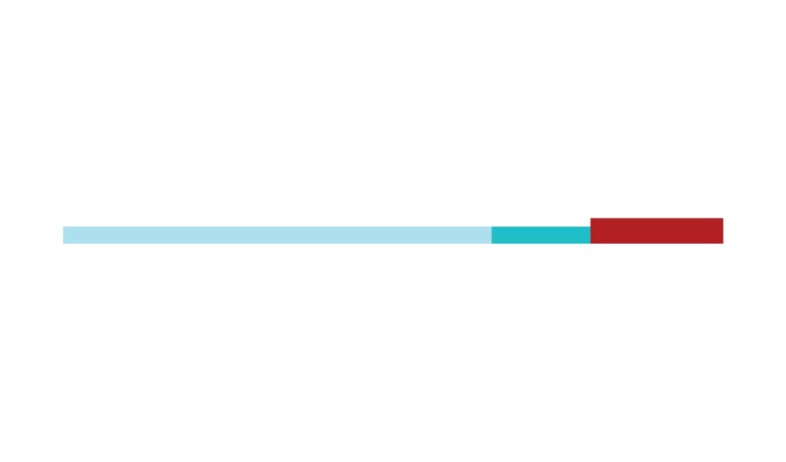
Roe et al.	0	Neurology	**2007**	0.01	75.746	**2008**	2012	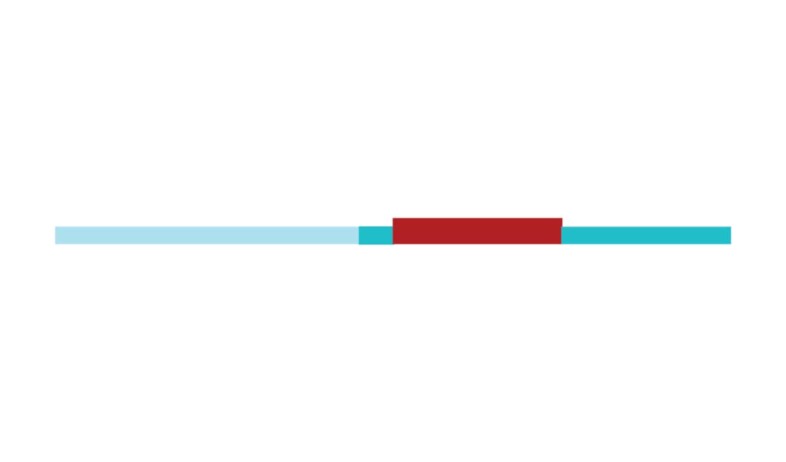
Jones et al.	0	J Int Neuropsych Soc	**2011**	0.01	70.744	**2015**	2017	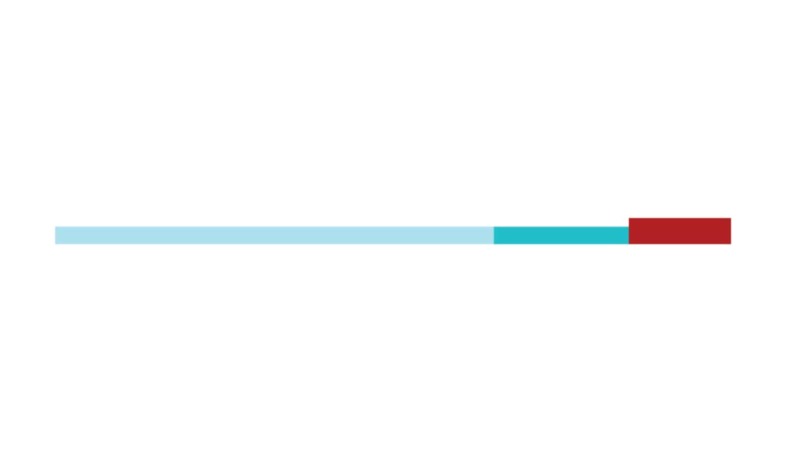
Stern	0	Neuropsychologia	2009	0.05	54.507	**2012**	2014	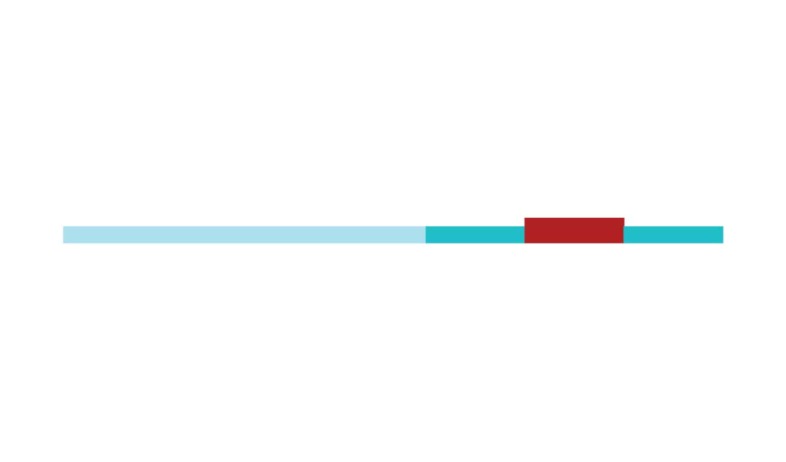
Valenzuela and Sachdev	0	Psychol Med	2006	0.08	54.275	**2014**	2015	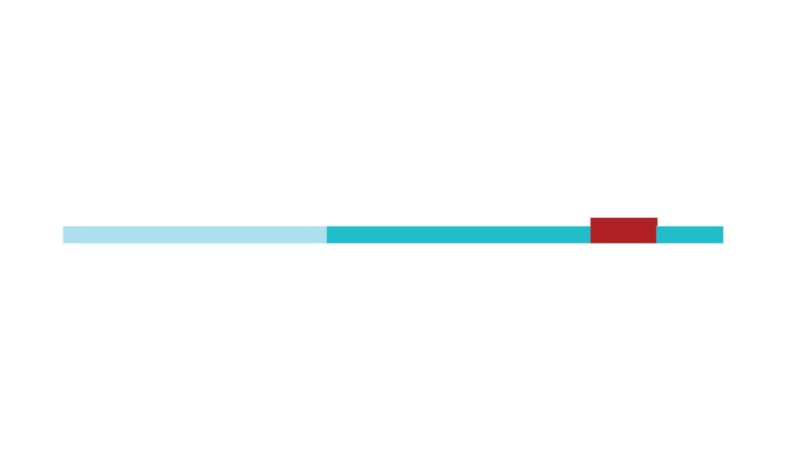
Rentz et al.	0	Ann Neurol	2010	0.01	42.871	**2011**	2015	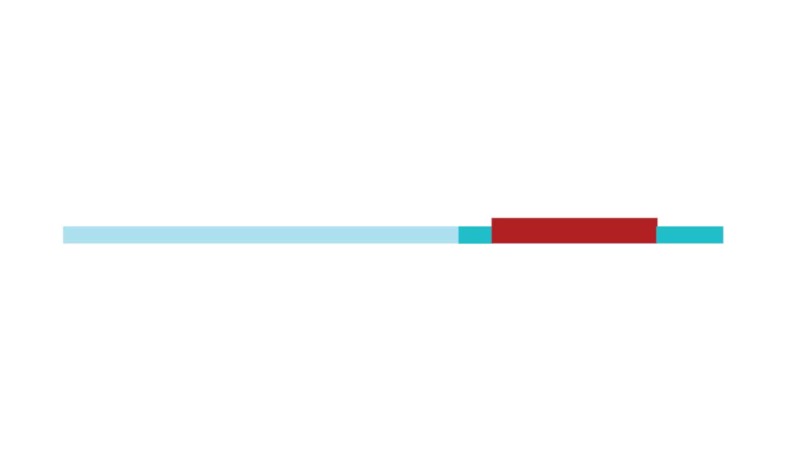
Scarmeas and Stern	0	Neurology	2001	0.06	40.768	**2003**	2007	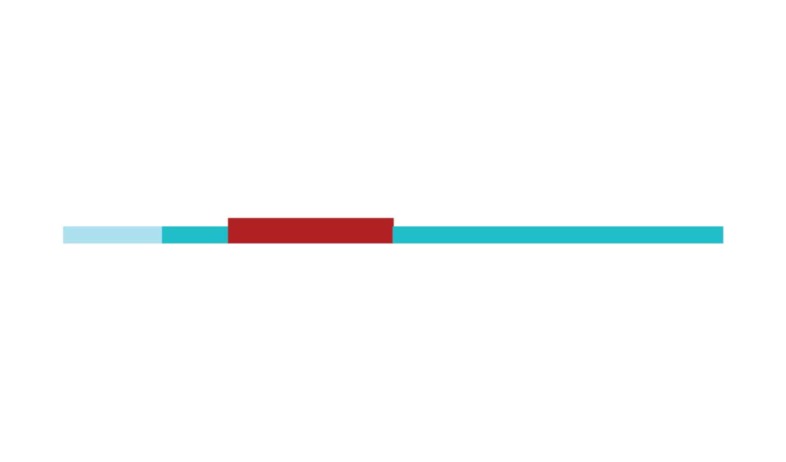
Alexander et al.	1	Am J Psychiat	1997	0.01	89.673	**1999**	2010	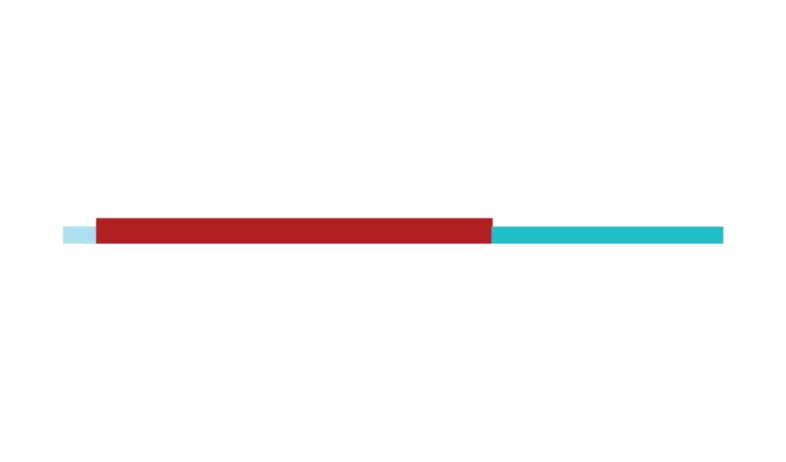
Roe et al.	1	Arch Neurol-Chicago	2008	0.01	71.659	**2010**	2011	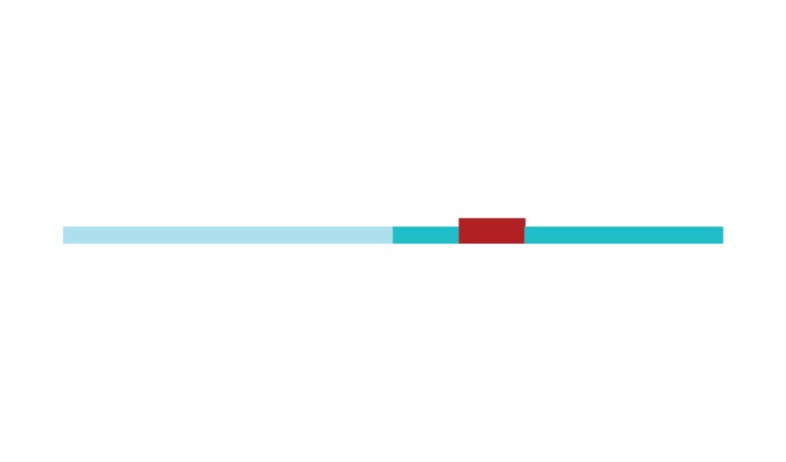
Stern et al.	1	Ann Neurol	1992	0.22	61.442	**2003**	2007	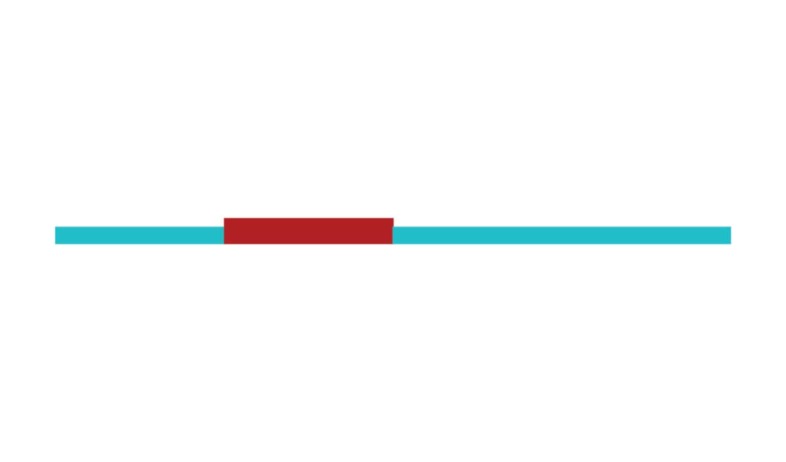
Katzman et al.	1	Ann Neurol	1988	0.13	42.944	**2005**	2013	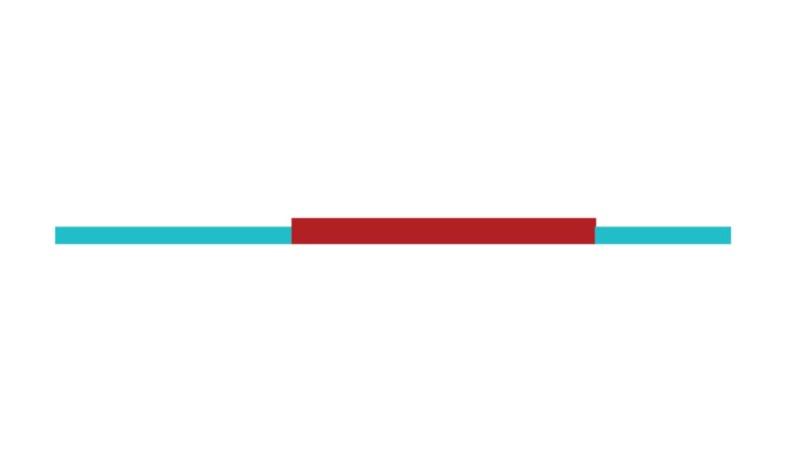
Schweizer et al.	2	Cortex	2012	0.01	84.039	**2014**	2017	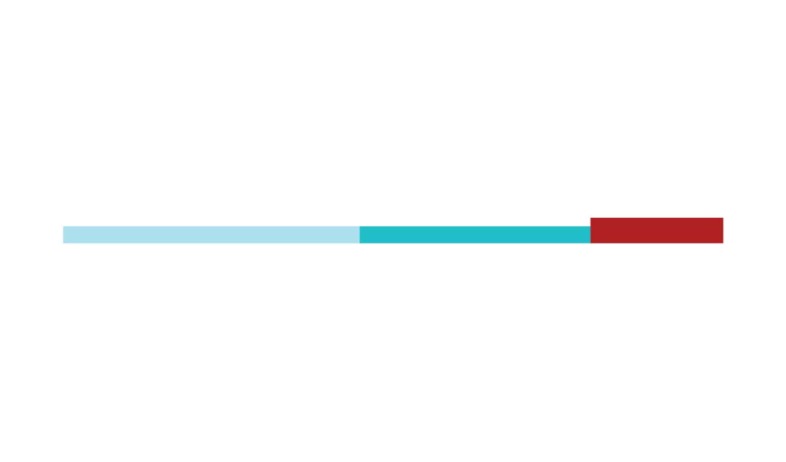
Bialystok et al.	2	Neuropsychologia	**2007**	0.22	78.819	**2013**	2017	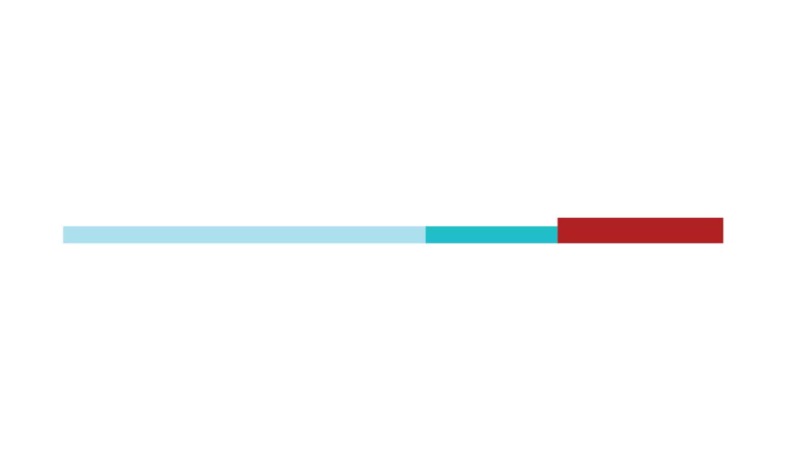
Chertkow et al.	2	Alz Dis Assoc Dis	2010	0.11	76.964	**2014**	2017	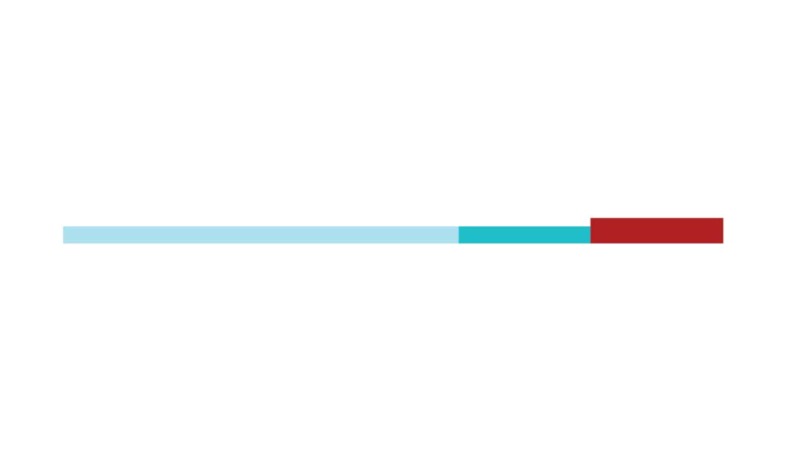
Gollan et al.	2	Neuropsychologia	**2011**	0.05	69.731	**2015**	2017	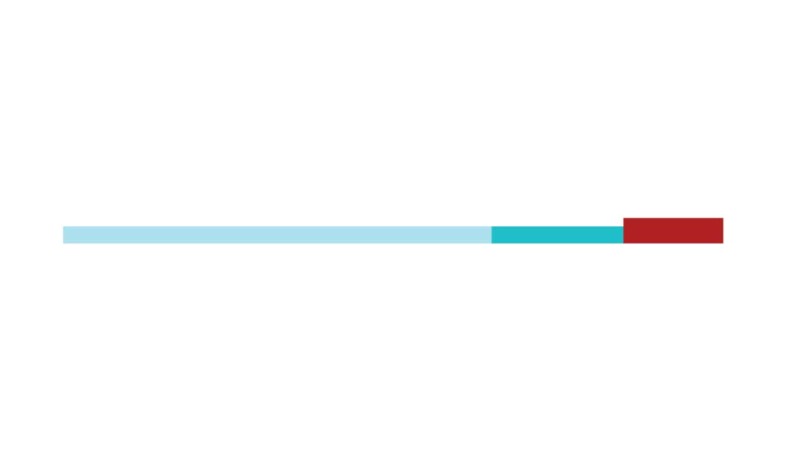
Craik et al.	2	Neurology	2010	0.03	63.890	**2014**	2017	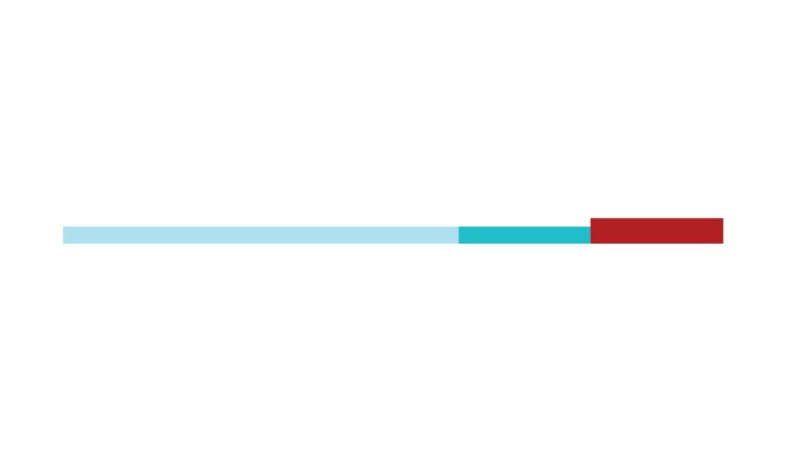
Stern et al.	3	Neurology	**1999**	0.24	103.659	**2001**	2009	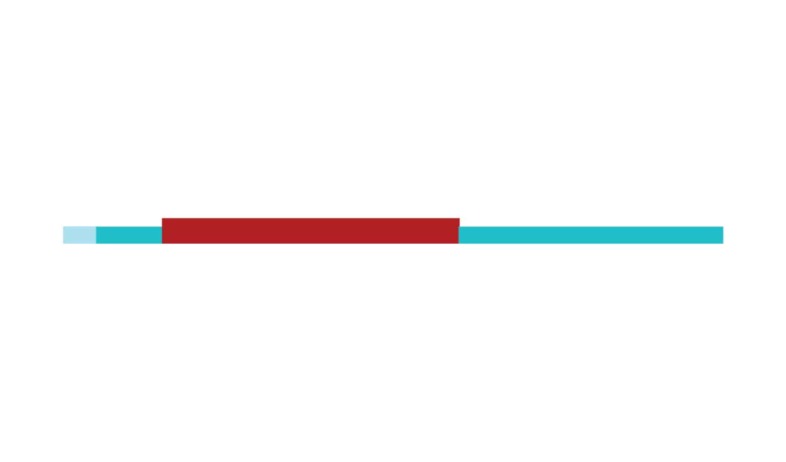
Barulli and Stern	3	Trends Cogn Sci	2013	0.01	52.912	**2015**	2017	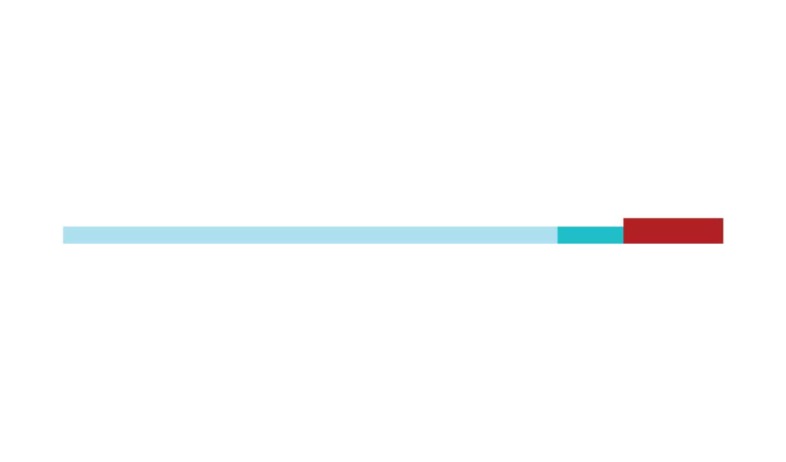
Satz	4	Neuropsychology	1993	0.02	100.073	**2003**	2011	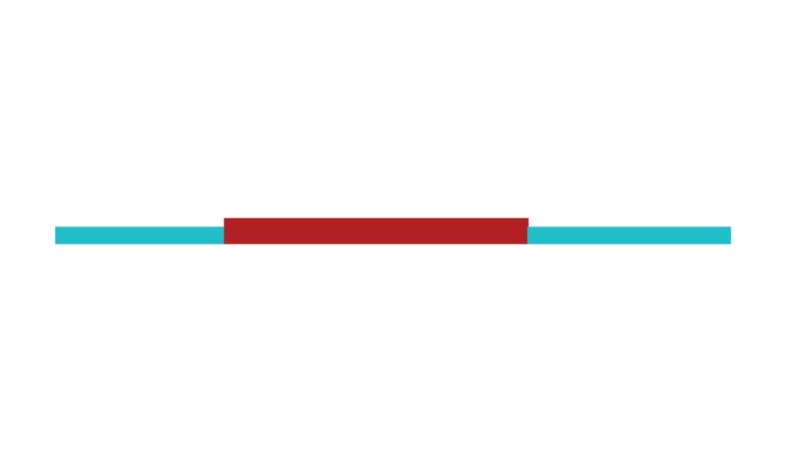
Meng and D'Arcy	4	Plos one	2012	0.04	84.293	**2014**	2017	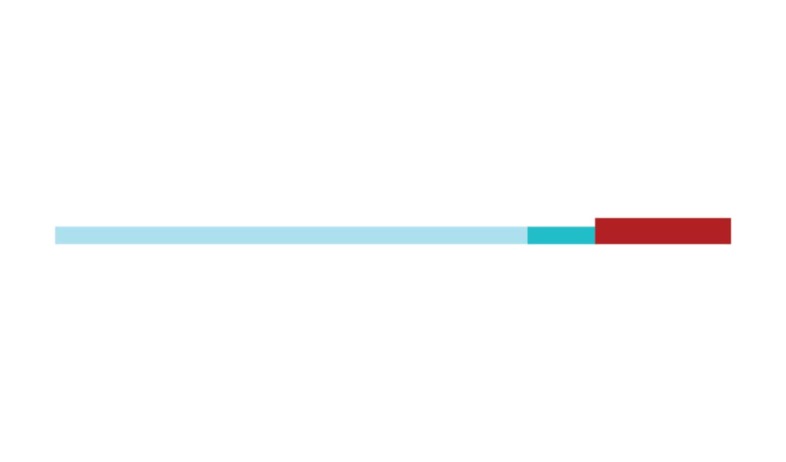
Wilson et al.	4	Jama-J Am Med Assoc	**2002**	0.01	78.773	**2010**	2013	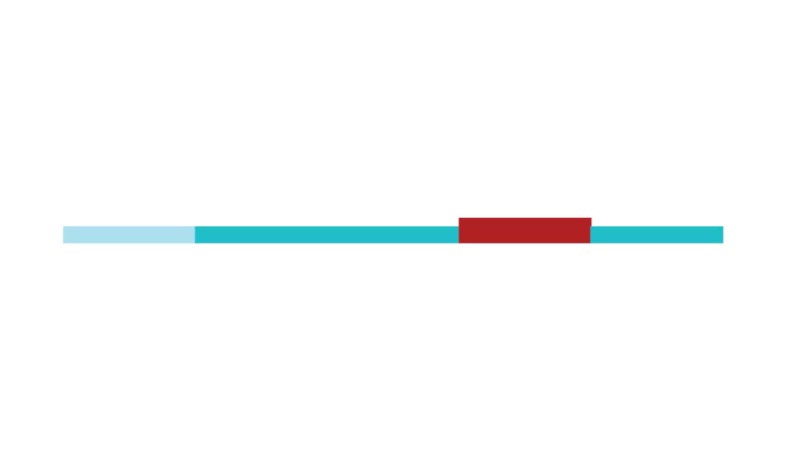
Fratiglioni and Wang	4	J Alzheimers Dis	**2007**	0.03	56.810	**2012**	2013	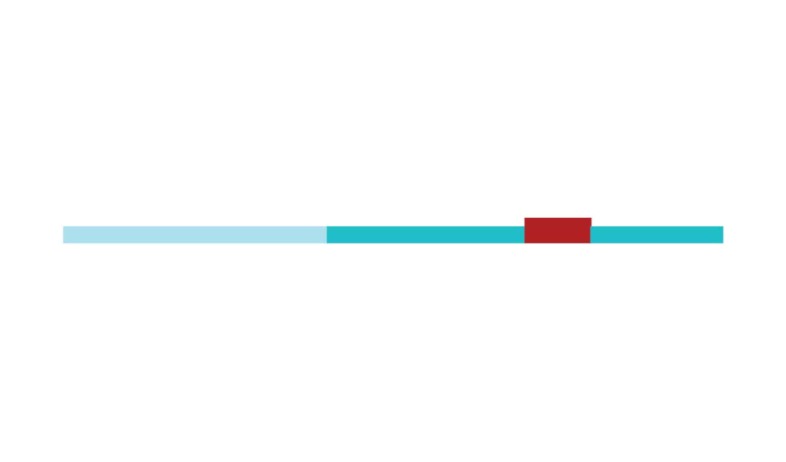
Valenzuela and Sachdev	4	Psychol Med	**2007**	0.08	42.538	**2013**	2017	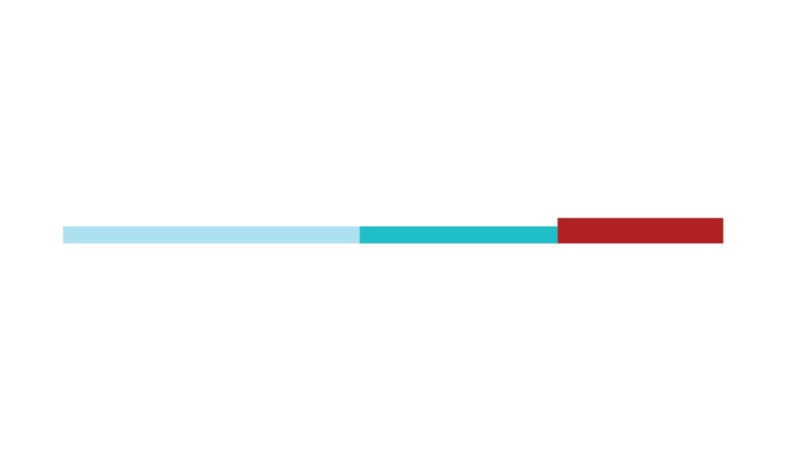

Source: authors (2018) from CiteSpace.

### Most active clusters and references

A citation burst can be used to detect the most active articles and areas of research. A citation burst provides evidence that a publication is associated with a surge in citations, attracting an extraordinary degree of attention from the scientific community.^61^ All clusters from #0 to #4 have bursts of citations, meaning they are representative of the diversity of interests in the field of research. Citation bursts provide a useful means of tracking the development of research areas. Cognitive reserve (#0) has the articles with strongest citation bursts, meaning that mainly after 2002, the beginning of the burst, the major efforts of the research field concentrated in this area of research. Other research interests, mainly after 1999, involve functional ability (#1) and mortality data (#3) then, after 2003, on reserve mechanisms (#4); and after 2013 in executive control (#2). Table 3 shows the most active areas of research and articles, displayed by burst strength per cluster.

### Emerging trends in the network of CR and dementia

The intellectual structure of the knowledge of co-cited references can be measured by modularity.^13^ Newly published articles may have little or no impact on the structure of the network, or may create a profound structural change, when deep changes occur in the modularity. Figure 3 shows the change in modularity during the 1998-2017 period. It is notable that modularity dipped mainly in 1999, 2002, 2007, and 2011. Therefore, we investigated potential emerging trends looking at publications with bursts of citations in these years, because we expected them to play an important role in changing the overall intellectual structure of CR and dementia. The top publications are marked with shading in Table 3, and include:

In 1999, Stern^63^ focuses on the effect of level of education and occupational attainment on memory in AD patients.

In 2002, Wilson et al.^64^ focuses on the effect of frequency of participation in cognitively stimulating activities on the risk of AD.

In 2007, the three relevant articles are: Fratiglioni & Wang^65^ focuses on the effects of education, adult-life occupational work complexity, mentally and social integrated lifestyle in late life, as factors to affect the onset of clinical dementia and AD; Bialystok et al.^62^ focuses on the effect of lifelong bilingualism on dementia in old age; and Valenzuela & Sachdev^66^ centers on LEQ as a tool for estimating brain reserve in older individuals.

In 2011, the three relevant articles are: Tucker & Stern^6^ focuses on the effect of executive functions task on CR; on the association between CR and neural efficiency, capacity, and ability; and on the fact that CR is not fixed but continues to evolve during the lifespan; Gollan et al.^67^ focuses on the effects of bilingualism on CR and suggested an upper limit on the extent to which reserve can delay dementia; and Jones et al.^68^ focuses on approaches for quantifying reserve using latent variable models, with an emphasis on their application in the analysis of data from observational studies.

## CONCLUSION

This bibliometric study carried out during the 1998-2017 period has allowed a number of conclusions to be drawn in line with the objective established in the introductory section of this paper. The exploration of the literature on CR and dementia from WoS databases has outlined the evolutionary trajectory of the collective knowledge over the past twenty years and highlighted the areas of active pursuit and future research. Based on the network visualization and document co-citation analysis using CiteSpace and VosViewer, this study evaluated emerging trends and patterns of publications, citations, journals, institutions, and areas of research in the literature. The top clusters covered a range of interests, reflecting the interdisciplinary nature of CR and dementia. They are foundational to the field of research.

To the best of our knowledge, this study represents the first attempt to apply CiteSpace to explore and visualize CR and dementia knowledge. It is one of only a few investigations that have focused on co-citations as a marker of development of this domain from different perspectives. The findings of the present investigation demonstrate the potential of bibliometric visualization techniques for studying the scientific literature.

First, by visualizing the relational analysis of top authors and articles, the study provides insights into patterns of international research focus. The clustering technique used in this work identifies key articles that share similar topics. Articles that serve as an important bridge between two clusters are also detected in the network, namely Stern^5^^,^ 58 ^,^ 59 and Bialystok et al.^62^ Secondly, the bibliometric visualization used in this paper provides important temporal data by displaying nodes in different colours. A longitudinal view of cluster analysis and citation bursts of key articles adds a new dimension to the analysis and provides insights into the flow of major trends and collaborations. These temporal data allow researchers to identify research frontiers by highlighting emergent hot topics, authors, and articles.^41^

Thirdly, co-citation analysis is a useful method of providing insights into a field based on a large sample of documents.^41^ Multiple metrics help elucidate and explore relationships between articles and citations. Betweenness centrality can reflect the potential pivotal point and new ideas in the CR and dementia field; while density, modularity, and burst strength provide a more objective metric analysis of the network.

Another contribution made by the research is the understanding it promotes regarding the way knowledge is structured in the field of CR and dementia. The study of clusters, previously described, showed that cluster #1, cluster #3, and cluster #4, due to bursts of citations, are the major current areas of research. Consequently, the maturity of research on this domain is evident by the fact that it has shifted from being focused mainly on CR to more specific aspects with a broader disciplinary base (functional ability, #1; mortality data, #3; reserve mechanisms and executive control, #4). Therefore, this field of study is becoming more multidisciplinary, increasingly being analyzed from the different angles provided by diverse scientific approaches, which complement and enrich its content. In terms of authors’ affiliations, among the top universities, the leader is located in the USA: Harvard University followed by Colombia University and UCLA. The network of journals shows a central-peripheral structure, where Neurology and Journal of Alzheimer Disease are ranked first, followed by Journal of International Neuropsychological Society. The investigation of the most cited articles has allowed the mapping of the intellectual structure of this field. Hence, the potential for development of the field of CR and dementia as a research focus is immense.

The study has some limitations. First, this work was restricted to English language journals. It is likely that some of the literature may have been published in other languages. Secondly, despite the relevance of the WoS databases to CR and dementia research, other important studies could have been included within other databases. Nevertheless, it is evident that bibliometric analysis has helped to characterize, both qualitatively and quantitatively, the CR and dementia research field in terms of its development, trends of investigation and collaboration networks. As a result, researchers have been equipped with new tools of exploration.

The results help further understanding on the intellectual structure of this field through an innovative methodology, using co-citation analyses to understand the development of CR and dementia from different perspectives. The study findings demonstrate the potential of bibliometric visualization techniques to investigate the scientific literature.
